# Dr. Skinner’s F. C. High Potencies

**Published:** 1884-04

**Authors:** 


### DR. SKINNER'S F. C. HIGH POTENCIES.

Through the kindness of Dr. Skinner we are in a position to supply the whole of his high potencies—a complete set, carriage paid to New York. Price on application. Alfred Heath & Co., Homœpathic Chemists, 114 Ebury Street, London, England, makers of English fresh plant tinctures. Special low rates to buyers in bulk in the spring.

